# Evaluation of in vitro and in vivo anticancer potential of two 5-acetamido chalcones against breast cancer

**DOI:** 10.17179/excli2017-624

**Published:** 2017-10-19

**Authors:** Sonal Wankhede, Nitesh Kumar, Lalitha Simon, Subhankar Biswas, Karthik Gourishetti, Grandhi Venkata Ramalingayya, Mit Joshi, C. Mallikarjuna Rao

**Affiliations:** 1 Department of Pharmacology, Manipal College of Pharmaceutical Sciences, Manipal University, Manipal, Karnataka-576104, India; 2 Department of Chemistry, Manipal Institute of Technology, Manipal University, Manipal, Karnataka-576104, India

**Keywords:** chalcones, breast cancer, N-methyl-N-nitrosourea

## Abstract

Two 5'acetamido chalcones, C1 and C2 were synthesized by Claisen-Schmidt condensation method and characterized by IR, LC-MS, ^1^H NMR and ^13^C NMR. These compounds were evaluated for anticancer activity *in vitro* in breast cancer cell lines (MCF-7 and MDA-MB-231) using MTT assay, anti-metastatic assay, apoptotic screening by AO/EB staining and *in vivo* in N-Methyl-N-nitrosourea (MNU) induced breast carcinoma model. Sprague-Dawley rats with developed tumors (50 mg/kg MNU *i.p.*) were grouped in four, namely MNU control (0.25 % of CMC *p.o.*), standard group (doxorubicin 2 mg/kg once in 4 days, *i.p.*), C1 and C2 groups (50 mg/kg *p.o.* each). After 21 days of treatments, tumor volume and weight were assessed. Excised tumors were subjected to DNA fragmentation study. MTT assay showed IC_50_ values of 62.56 and 37.8 µM by for C1 and C2. Both compounds increased apoptotic bodies more than 3 fold compared to normal control in AO/EB staining. Antimetastatic (scratch wound) assay showed a significant (p<0.05) reduction in cell migration after 24 h and 48 h treatments compared to normal control. In *in vivo* studies, tumor weight and tumor volume were significantly (p<0.05) reduced in the doxorubicin group as well as in test groups compared to MNU control. DNA fragmentation assay showed an increase in the number of bands formed in C1 and C2 compared to normal control. Results obtained from *in vitro* and *in vivo* studies demonstrated the significant anticancer potentials of C1 and C2.

## Introduction

Cancer is a threat of 21^st^ century and is the 2^nd^ most common cause of death after cardiovascular diseases (Cooper and Hausman 2000[5]; Global Burden of Disease Cancer 2015[10]). It is an outcome of abnormalities in normal cell behavior characterized by uncontrolled proliferation, dedifferentiation, invasiveness, metastasis and alteration in several other biological processes that maintain the normal cycle of life. Among the various types of cancers breast cancer is the most common type of cancer in women. Current medication for breast cancer includes either alone or combination of approaches such as surgery, radiation therapy, chemotherapy, hormone therapy, targeted therapy etc. Except for surgical therapy, all the above-mentioned approaches of treatments have their own shortcomings such as mouth soreness, nausea, vomiting, weakness, loss of appetite, fatigue, hair loss etc. (Carelle et al. 2002[4]). These side effects not only alter the quality of life of survivors but sometimes lead to their mortality. 

Along with higher efficacy, chemotherapeutic drugs have well established side effects in breast cancer. Thus, extensive research is required in this area to hunt for newer molecules that can provide better therapy without compromising the quality of life. Chalcones are one such category of compounds, which can be thought in this perspective. They are the precursors of flavonoids and flavonol and are also present in edible plants. Being a naturally occurring component, chalcones are considered to have fewer side effects like genotoxicity as seen with other chemotherapeutic agents (Kumar et al. 2003[15]) Several chalcones have been synthesized which showed very potent cytotoxicity against several cancer cell lines like MDA-MB-231, MCF-7 etc. with established mechanism of action like induction of cell cycle arrest and apoptosis (Kumar et al. 2003[15]). However, there are very few studies available on acetamido chalcones as an antitumor agent. In view of above scenarios in breast cancer and related therapies, our study was focused on evaluating *in vitro *& *in vivo *anti-cancer activity of 5'-acetamido chalcones against breast cancer. The study was focused on the synthesis and characterization of compounds, evaluation of its *in vitro* and *in vivo *anticancer potential in breast cancer model and assessment of its antioxidant property. 

## Materials and Methods

### Materials

All the chemicals required for different experiments were procured through Manipal College of Pharmaceutical Sciences (MCOPS), Manipal University (MU) from designated manufacturers/suppliers. 2,2'azino-bis(3-ethylbenzothiazoline-6-sulphonic acid (*ABTS)*, 2,2-Diphenyl-1-picrylhydrazyl (DPPH), (5,5'-dithio-bis-[2-nitrobenzoic acid]) DTNB, Dulbecco's Modified Eagle's Medium (DMEM), Dimethyl sulfoxide (DMSO) were all procured from Sigma-Aldrich, Acetic acid, Hydrochloric acid (HCL), Methanol, MTT reagent, propidium iodide were all purchased from HiMedia laboratories, Sodium hydroxide (NaOH), Sodium chloride (NaCl), Sulphuric acid (H_2_SO_4_) was obtained from SD Fine Chemicals, Foetal bovine serum (FBS) was purchased from Invitrogen. Cell Lines: MCF-7 and MDAMB-231 were obtained from National Center for Cell Sciences (NCCS) Pune.

#### Synthesis of chalcones (different functional group substituted) 

Claisen-Schmidt condensation reaction was followed for the synthesis of required chalcones (Simon et al. 2016[21]). Briefly, paracetamol was weighed and transferred into a 100 mL round bottom flask. Anhydrous aluminium chloride and nitrobenzene were added to the flask followed by gradual addition of acetyl chloride. The temperature was maintained at 130 °C for three hours. After the reaction is complete, the reaction mixture was poured into a mixture of crushed ice and concentrated hydrochloric acid with vigorous stirring and filtered. The crude product thus obtained was first washed with water and then with toluene and crystallized following which light brown needle-shaped crystals were obtained.

**Figure 1:**
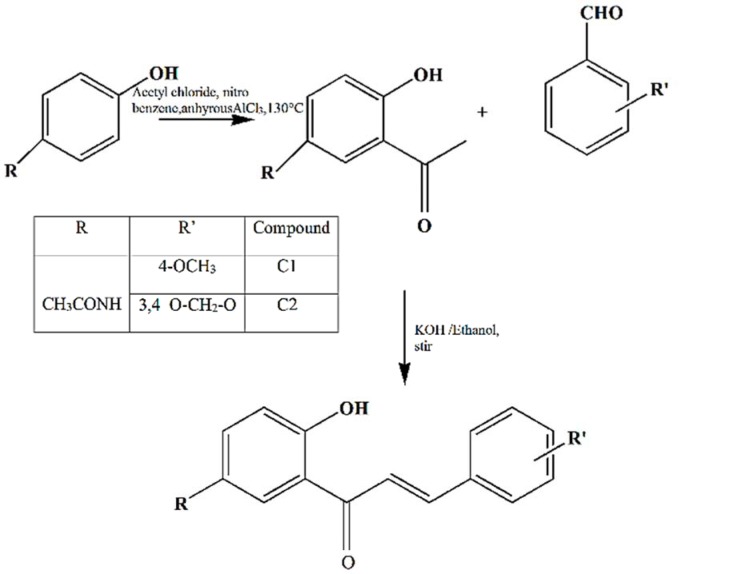
Synthesis of 5'-acetamido-2'-hydroxychalcones

The product from the above step was dissolved in ethanol and 4-methoxy/3,4-methylenedioxy benzaldehyde was added to it. To this, 10 % aqueous KOH solution was added and the reaction mixture was stirred around 30 °C for about 12 h, and poured into crushed ice containing 5N HCl. The precipitated product was washed with cold water, dried and recrystallized from ethanol to yield dark yellow crystals (Figure 1(Fig. 1)).

Thin layer chromatography (TLC) technique was applied using hexane and acetone as a solvent system in a ratio of 6:4 for monitoring of the completion of the reaction. The retention factor was calculated to check the reaction completion as well as for establishing the purity of the synthesized compound. After the reaction completion % yield was calculated. The melting point for each compound was determined using melting point apparatus. Mass spectroscopy was used to determine the molecular weight of the compounds. The instruments used in the present study was Thermo Scientific^TM ^Q Exactive ^TM^ Focus hybrid quadrupole-Orbitrap MS, by Thermo Fischer Scientific Corporation, USA. IR spectroscopy was used for structural confirmation in the infrared region of the electromagnetic spectrum using Shimadzu FTIR, by Shimadzu Corporation, Japan. NMR spectroscopy was used to determine the number of protons (^1^H) and carbon atoms (^13^C) present in the compound to confirm its molecular structure. The instrument used was Bruker NMR by Bruker Corporation, USA.

#### In vitro cytotoxicity screening

MTT assay is a colorimetric assay for assessing cell viability. Cells were seeded in sterile 96-well tissue culture plate. Cells were allowed to attach for 24 h. The compound was prepared before the beginning of the experiment and serially diluted with suitable medium to get the different concentrations (50-500 μM). After 24 h, cells were treated with 100 μl of test compounds from respective top stocks for 48 h. After 48 h 50 μl of MTT reagent (Stock: 2 mg/ml in PBS) was added in each well and incubated for 3 h at 37 °C. The cells in the control group did not receive treatment. Each treatment was performed in triplicates. After 3 h of incubation medium along with MTT was aspirated and 200 μl of 100 % DMSO was added to each well to solubilize formazan crystals. The Optical density (O.D) was measured by an ELISA plate reader at 540 nm. Percentage viability of each compound was calculated (Gerlier and Thomasset, 1986[9]).

#### Acridine orange/ethidium bromide staining (AO/EB staining) 

Chromatin condensation and nuclear fragmentation are one of the important apoptotic hallmarks. Therefore, examination of the morphology of the cells is very important. Cells were harvested and (5 × 10^4^) cells were seeded in 12 well plate and allowed to attach overnight. Cells were treated with selected test compound at their IC_50_ (determined from MTT assay) for 48 h incubation. Media was aspirated and each well was washed with PBS. Cells in each well were fixed with ice-cold ethanol for 20 min. Next, ethanol was aspirated from each well. Acridine orange (10 mg/ml) and ethidium bromide (10 mg/ml) stain was prepared in PBS and mixed in the ratio of 3:2 and added to each well. After 20 min, unbound stain was washed using PBS solution and observed under fluorescent microscope (NikonTS100F) with an excitation of 450-490 nm (Ribble et al. 2005[19]).

#### In vitro anti-metastasis assay 

Cells, grown in DMEM containing 10 % FBS, were seeded into 6 well plate for 24 h to a 70-80 % confluent monolayer. Using a sterile 200 μl pipette tip, a monolayer was scratched across the center of the well. The well was washed twice with medium to remove detached cells and replenished with fresh medium containing test compounds. The cells were washed twice with PBS. Images were taken at 0 h (immediately after wounding), 24 h and 48 h of treatment at 40X magnification using an inverted microscope. (Bishayee et al., 2013[2]).

#### Antioxidant assay by DPPH assay method

The antioxidants react with 1,1-diphenyl-2-picrylhydrazyl radical (DPPH), a stable free radical and convert it to 1,1-diphenyl-2-picryl hydrazine. The ability to scavenge DPPH was measured at an absorbance of 517 nm. To the 96 well plates, 50 μl of various concentrations of the compound was added in duplicates. Followed by 50 μl solution of DPPH (0.1mM) was added using a multichannel pipette. Ascorbic acid was used as the standard for comparison. Control included an equal amount of ethanol and DPPH. After 20 minutes incubation in the dark, absorbance was recorded at 540 nm. The experiment was performed in triplicate (Bishayee et al., 2013[2]).

% scavenging= [(*ABS control - Abs test*)] / (*Abs control*) x 100

### In vivo study

#### Animals

Sprague-Dawley rats were maintained as per the guideline laid by Committee for the Purpose of Control and Supervision of Experiments on Animals (CPCSEA), Govt. of India. Briefly, animals were acclimatized in an experimental room at temperature 23 ± 2 °C, controlled humidity and 12:12 hour light and dark cycle. They were provided with food and water *ad libitum*. Study was conducted after obtaining ethical committee clearance from the Institutional Animal Ethics Committee of KMC, Manipal (Clearance No. IAEC/KMC/ 73/2015).

#### Acute toxicity study

Acute toxicity study will be conducted according to OECD 425 guideline with 5 female mice in each group. 2000 mg/kg dose was given orally to each mouse and the animals were observed for 14 days. No motility was observed with the test compound at a dose of 2000 mg/kg (Botham, 2004[3]).

#### In vivo antitumor activity in MNU-induced breast cancer model

Breast tumor was induced in Sprague-Dawley rats by injecting 50 mg/kg MNU intraperitoneally (McCormick et al., 1981[17]). After the development of tumor i.e., 90 days from the injection, the animals were grouped in the followings: MNU control group: animals were administered with 0.25 % of CMC (*p.o*) & 10 ml/kg saline (*i.p*). Standard group: animals were treated with doxorubicin at a dose of 2 mg/kg once in 4 days (*i.p*.). C1 group: animals were treated with C1 at a dose of 50 mg/kg (*p.o.*). C2 group: animals were treated with C2 at a dose of 50 mg/kg p.o. Animals were administered with the aforesaid compounds for 21 days. Tumor volume was monitored every 6 to 7 days using a digital Vernier caliper. The length (a) and width (b) of tumor were measured at right angles. The tumor volume was estimated using Carlson's formula V=4/3Π a^2^b, where 'a' and 'b' represent the longest and shortest tumor radii respectively (Kumar et al., 2014[14]). On day 22^nd^, blood was withdrawn, the tumor was isolated and various organs were isolated. The isolated tumor was subjected to DNA fragmentation study, histopathological study and endogenous antioxidant estimation.

#### Organ index

Organs namely, liver, kidney, heart and spleen were isolated and weighed in grams. The organ index was calculated by the following formula (Fidler, 2003[8]): 

Organ index = weight of organ in grams / body weight of animals in grams

#### Hematological parameters 

Following parameters were monitored in breast cancer-bearing rats to detect the influence of chalcones: White blood cell count, Red blood cell count Haemoglobin count, Platelets count, Percentage of lymphocytes, monocytes and granulocytes using ERMA veterinary blood cell counter, Japan. 

#### DNA ladder assay

For the apoptotic study, the electrophoretic analysis of fragmented DNA was done; DNA was isolated from the tumor samples. 100 mg of tumor samples were homogenized in 1 ml of lysis buffer [20 mM Tris (pH 7.5), 0.15 M NaCl,1 mM EDTA, 1 % SDS] then centrifugation at 3000 g for 10 min was done at RT and a supernatant was aspirated out. 20 µL Proteinase K (stock 20 µg/ml) was added to the supernatant and incubated for 2 hours at 37 °C. This was then centrifuged at 12000 g for 10 min at RT. The supernatant was collected in a new tube; the DNA was then precipitated by adding chilled absolute ethanol and 0.1 M NaCl. The precipitated DNA was collected by centrifugation and washed with 70 % ethanol. The DNA pellet was dried and dissolved in distilled water. The DNA samples were prepared in a loading solution (0.25 % bromophenol blue, 0.25 % xylene cyanol FF and 30 % glycerol) in the ratio 1:5. 10 µL of the DNA sample was loaded into each well of 1.8 % agarose gel containing 0.5 mg/ml ethidium bromide. The electrophoresis was carried out in TAE buffer for 1.5 hours. The DNA bands in the gel were observed and photographed in a Syngene G-Box Chemi XRQ Gel documentation system (Kuo et al., 1996[16]).

#### Histopathological examination 

All the animals of MNU control group, standard group, C1 and C2 treated groups were sacrificed on day 24 from the initial day of sensitization by light ether anesthesia followed by carotid bleeding. A small portion of the tumor was selected for histopathology. The specimens were stored in 10 % neutralized buffered formalin, and processed for histopathological findings.

#### Estimation of endogenous antioxidants

 Liver and tumor homogenate (10 %w/v) were prepared with ice-cold phosphate buffer using Teflon-glass homogenizer. This homogenate was centrifuged at 10,000 rpm for 10 min, the pellet was discarded and the supernatant was used for the estimation of Total protein: (Goa, 1953[11]), Superoxide dismutase estimation (SOD) (Hamberg et al., 1968[12]), Lipid peroxidation (TBARS) estimation (Devasagayam et al., 2003[6]), and Estimation of reduced glutathione (GSH) (Anderson, 1985[1]). Serum was used for evaluation of the safety parameters on the liver by estimation of ALT, AST and on the kidney by creatinine estimation using the semi-automatic auto analyzer. 

## Results

### Synthesis and characterization of compounds

The method of synthesis for the compounds was Claisen Schmidt condensation reaction. The R_f_ value and % yield for C1 and C2 were found to be 0.388, 0.347 and 81.5 %, 92 % respectively. The purity of the compound was assessed by preliminary methods such as melting point and TLC. Further, the structural confirmation of purified C1 and C2 were performed by IR spectroscopy, LC-MS, ^1^H and ^13^C NMR which are as follows:

C1: Percentage yield 81.5%, Rf value-0.388, melting point160 ± 2 °C, IR (KBr cm^- 1^) 3443 (OH), 3250 (N-H stretch), 3053 (C-H aromatic), 1641 (C=O), 1616 (N-H bend), 1166 (C-O**^.^**), Mass-m/z (M+1) = 311.17; ^1^HNMR (400 MHz, DMSO) (D6) δ 2.039 (s, 3H, CH_3_), 3.835 (s, 3H, O-CH_3_), 6.94-8 (9H, Ar-H and H_α_, H_β_), 9.90 (s, NH), 11.96 (s, OH).

C2: Percentage yield 92 %, Rf value- 0.347, Melting point 198 ± 2 °C, IR (KBr cm^- 1^) 3443.05 (OH), 3342 (N-H stretch), 1650 (C=O), 1614 (N-H bend), 1150 (C-O), Mass-m/z (M+1) = 324.83; ^1^H-NMR (400 MHz, DMSO-D6) δ2.032 (s, 3H, CH_3_), 6.13 (s, 2H, O-CH_2_), 6.94-8.14 (8H, Ar-H and H_α_, H_β_), 9.88 (s, NH), 11.96 (s, OH); ^13^C-NMR: 400 MHz, (DMSO-D6) δppm: 24.13 (CH3), 102.28 (O-CH_2_), 107.47, 109.19, 118.14, 120.74, 121.39, 121.78, 126.43, 128.87, 129.39, 131.34, 145.05, 148.67, 150.41, 157.62, 168.55 (14C, Ar-C and C_α_,C_β_), 193.45 (C=O).

### In vitro evaluation

#### Preliminary in vitro cytotoxicity screening in human breast cancer cell lines

*In vitro* cytotoxic effect of synthesized compounds in MCF-7 & MDA-MB-231 by MTT assay after 48 h of exposure showed that molecules produced dose-dependent decrease in the percentage viability of the cells. IC_50 _values for C1 and C2 were found to be 62.56 μM and 37.8 μM respectively on MCF-7 cells and 21.19 μM and 12 μM on MDA-MB-231 respectively (Table 1(Tab. 1)). 

#### Acridine orange/ethidium bromide staining (AO/EB staining) 

**Figure 2:**
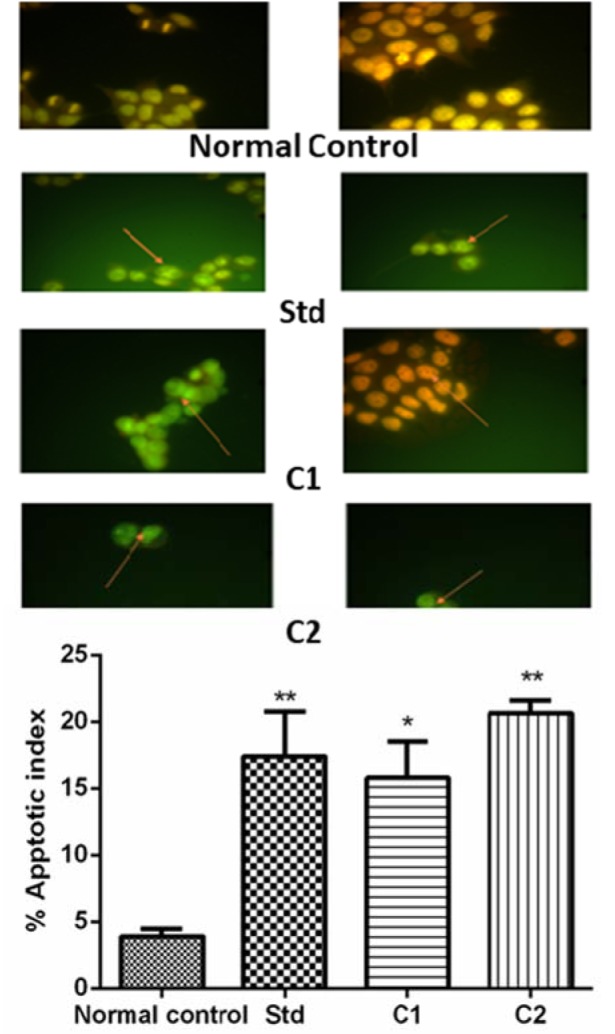
Acridine Orange/Ethidium Bromide staining of MCF-7. These arrows indicate the presence of an apoptotic cell. All the values are Mean ± SEM of three readings. Data are analyzed by One-way ANOVA followed by Tukey's post hoc method of analysis using GraphPad Prism, where *p< 0.05 compared to normal control

Apoptotic index significantly (p<0.05) increased in all treatment groups as compared to normal control. Doxorubicin, C1 and C2 showed apoptotic indices 17.41 ± 3.342, 15.79 ± 2.72520, 64 ± 0.95 respectively. The difference between apoptotic index of doxorubicin (2 mg/ml), C1 (50 mg/ml), and C2 (50 mg/ml), were not significant (p<0.05) compared to each other (Figure 2(Fig. 2)).

#### Scratch wound assay

**Figure 3:**
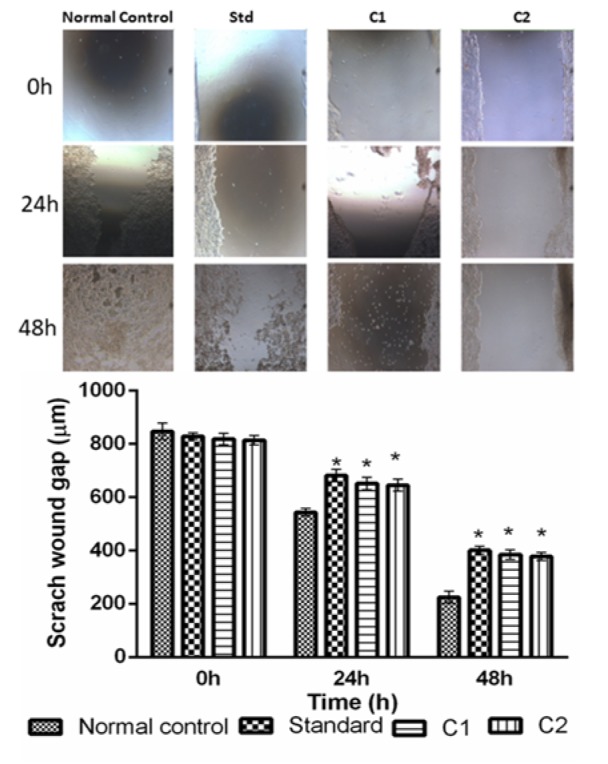
Effect of treatment C1 and C2 (50 µg/ml and 24 h and 48 h) on the healing of wound scratched in a monolayer of MCF -7 cells. All the values are mean ± SEM of three readings. Data are analyzed by Two-way ANOVA followed by Bonferroni post-test method of analysis using Graph Pad Prism, where *p < 0. 05 compared to normal control

Scratch wound assay was performed in metastatic human breast cancer cell line (MCF-7) to evaluate the potential of C1 and C2 to inhibit cell migration, metastasis, and anti-proliferative effect. Percentage of cell migration after treatment with molecules were evaluated by image J software. Two-time interval viz., 24 h and 48 h were selected to evaluate antiproliferative effect of drugs. Doxorubicin, C1 and C2 significantly (p<0.05) prevented the proliferation of cells compared to normal control at both time point (24 h, 48 h). However, no significant difference was observed among doxorubicin, C1 and C2 (Figure 3(Fig. 3)).

#### Antioxidant assay by DPPH assay method

The* in vitro *antioxidant activity of standard (Ascorbic acid), C1 and C2 were evaluated using DPPH assay method. All the tested compounds showed a dose-dependent decrease in the absorbance of the color of DPPH solution. IC_50_ values of C1, C2 and standard using DPPH assay was found to be 125.3, 83.94 and 32.42 μM respectively. C2 showed an IC_50_ value less than 100 μM, which could be considered as moderate antioxidant potential (Table 2(Tab. 2)).

### In vivo evaluation

#### Acute Toxicity Study

Compounds were found safe up to 2000 mg/kg *p.o. *following OECD 425 guideline of acute toxicity assessment.

### In vivo antitumor activity in MNU-induced breast cancer model

#### Effect of C1 & C2 on tumor volume

**Figure 4:**
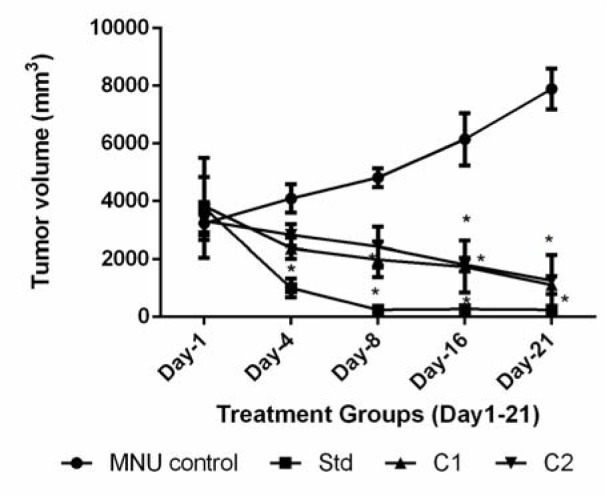
The effect of treatment C1 and C2 on tumor volume of rats in MNU induced breast cancer model. All values are mean ± SEM of six samples. Data are analyzed by Two-way ANOVA followed by Bonferroni post-test method of analysis using Graph Pad Prism, where *p < 0. 05 compared to MNU control

Tumor volume is a potential biomarker for breast cancer development. Day 4 onward, the doxorubicin-treated group showed significant (p<0.05) reduction in tumor volume as compared to MNU control. Day 8 onwards, treatment with C1 showed significant (p<0.05) reduction in tumor volume as compared to MNU control. Day 16 onwards treatment with C2 showed significant (p<0.05) reduction in tumor volume as compared to MNU control. On day 21, all the treatment group showed significant (p<0.05) reduction in tumor volume as compared to MNU control. Tumor volume was 243.67 ± 154.52 mm^3^ for standard, 1412.52 ± 309.55 mm^3 ^for C1 and 2760.99 ± 884.38 mm^3 ^ for C2, which were significantly (p<0.05) low as compared to MNU control (7890.69 ± 2707.97 mm^3^) on Day 21 (Figure 4(Fig. 4)).

#### Percentage reduction in tumor volume

**Figure 5:**
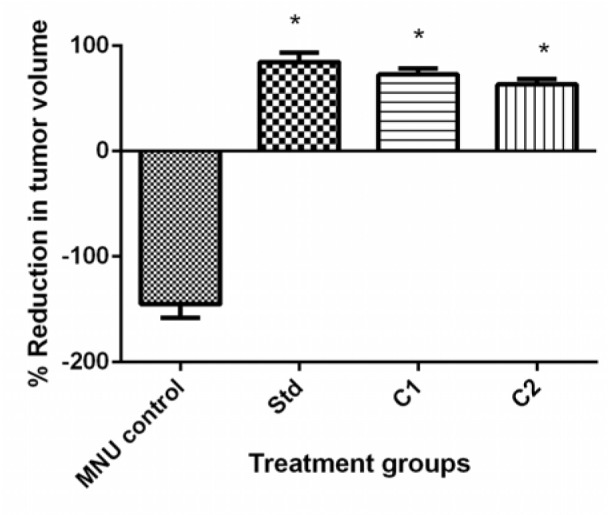
Effect of treatment C1 and C2 on percentage reduction in tumor volume on day 21 in comparison to day 1. All values are Mean ± SEM of six samples. Data are analyzed by One-way ANOVA followed by Tukey’s post hoc method of analysis using Graph Pad Prism, where *p< 0.05 compared to MNU control

Tumor volume is a potential biomarker for breast cancer development. The maximum reduction in tumor volume was seen in MNU control group. Treatment with C1 and C2 showed significant (p<0.05) percentage reduction in tumor volume as compared to MNU control. Doxorubicin showed significant (p<0.05) percentage reduction in tumor volume as compared to MNU control. Percentage reduction in tumor volume was 84.58 ± 9.167 for standard, 72.93 ± 5.646 for C1 and 63.47 ± 63.47 for C2, which were significantly (p<0.05) high compared to MNU control (-145.1 ± 12.91) (Figure 5(Fig. 5)).

#### Tumor weight

**Figure 6:**
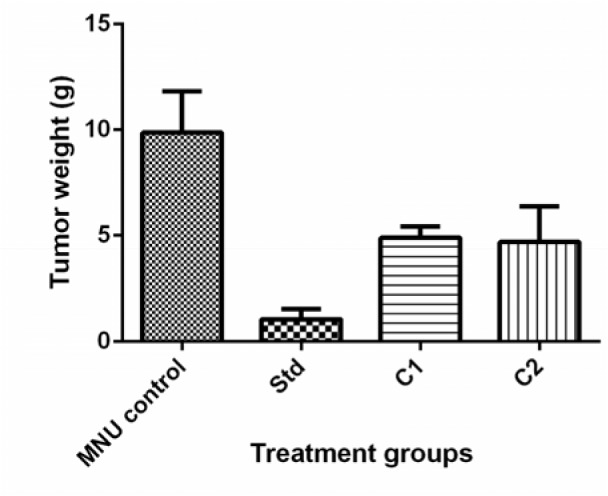
Effect of treatment C1 and C2 on tumor weight (g) of rats in MNU induced breast cancer model. All values are Mean ± SEM of six samples. Data are analyzed by One-way ANOVA followed by Tukey’s post hoc method of analysis using Graph Pad Prism, where *p< 0.05 compared to MNU control

Tumor progression can be determined by measuring tumor weight. All the treatment showed significant (p<0.05) reduction in tumor weight as compared to MNU control. Tumor weight was 9.87 ± 1.96 g for MNU control, 1.06 ± 0.49 g for standard, 4.90 ± 0.52 g for C1 and 4.70 ± 1.67 g for C2 (Figure 6(Fig. 6)).

#### Organ index

There was no significant difference observed in organ indices of liver, spleen, heart and kidney in any treatment group as compared to MNU control except doxorubicin (Standard), which showed a significant increase in the liver index as compared to MNU control (Table 2(Tab. 2)).

#### Hematological parameters

There was no significant difference observed in hematological parameters by the treatment of C1 and C2 as compared to MNU control. Doxorubicin treatment showed significant (p<0.05) decrease in the count of total RBC, WBC, and concentration of Hb, while a significant increase in platelets counts was observed when compared to MNU control.

The differential leucocyte counts were significantly (p<0.05) altered in the standard treatment group as compared to MNU control, except the % granulocyte level which was unaltered. In C1 and C2 treatment groups, no significant difference was observed as compared to MNU control group (Table 3(Tab. 3)).

### Effect of C1 & C2 on endogenous antioxidants

#### Estimation of endogenous antioxidants intumor

Standard, C1 and C2 showed significant (p<0.05) increase in SOD levels compared to MNU control in tumor samples. Glutathione levels were significantly increased in C2 treated group when compared with MNU control. There was a significant increase in MDA levels in standard (2 mg/kg) and C1 (50 mg/ kg) treated group when compared to MNU control group, while C2 did not produce any significant change (Table 4(Tab. 4)).

#### Estimation of endogenous antioxidants in Liver

Standard (Doxorubicin) showed significant (p<0.05) increase in SOD level as compared to MNU control in liver samples. Whereas no statistical difference was observed in C1 and C2 treatment groups as compared to MNU control. Glutathione levels were significantly (p<0.05) increased in Standard and C2 treated group compared with MNU control. MDA levels were significantly (p<0.05) increased by doxorubicin while significantly decreased by C1 treatment as compared to MNU control (Table 5(Tab. 5)).

#### DNA ladder assay

**Figure 7:**
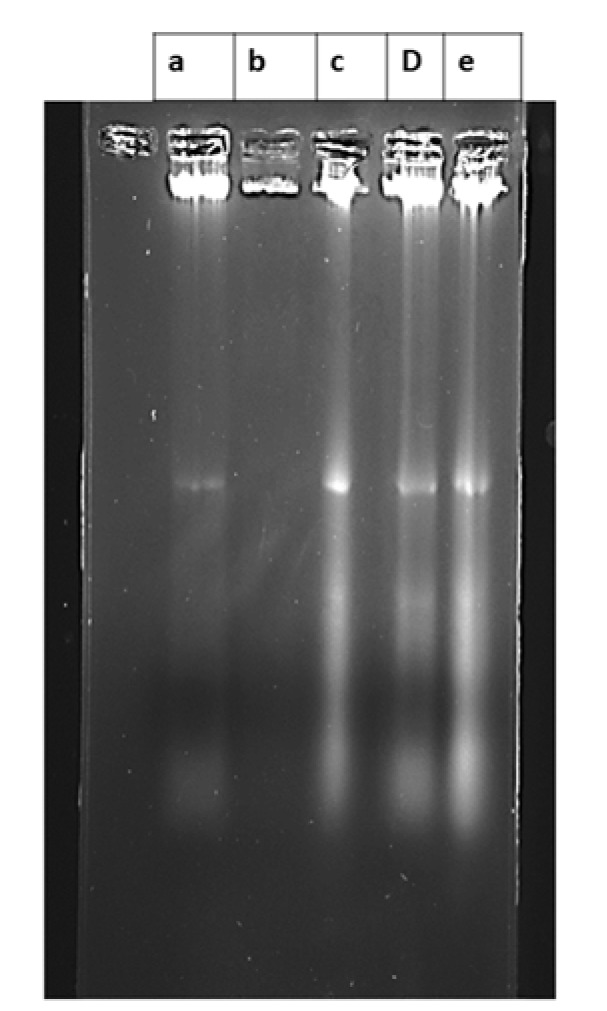
Effect of treatment on DNA ladder assay a= MNU control, b= std, c= C1, d= C2,e= C1

It was observed that intensity/no. of bands formed by C1 and C2 were more as compared to MNU control group in the excavated breast tumors. This is a direct evidence for the increased incidence of apoptosis in response to the treatments of C1 and C2. Standard did not show any change as compared to MNU control which might be due to improper DNA extraction from the tumors. Thus it may be stated that both C1 and C2 showed the incidence of apoptosis in breast tumors induced by MNU (Figure 7(Fig. 7)).

#### Histology

**Figure 8:**
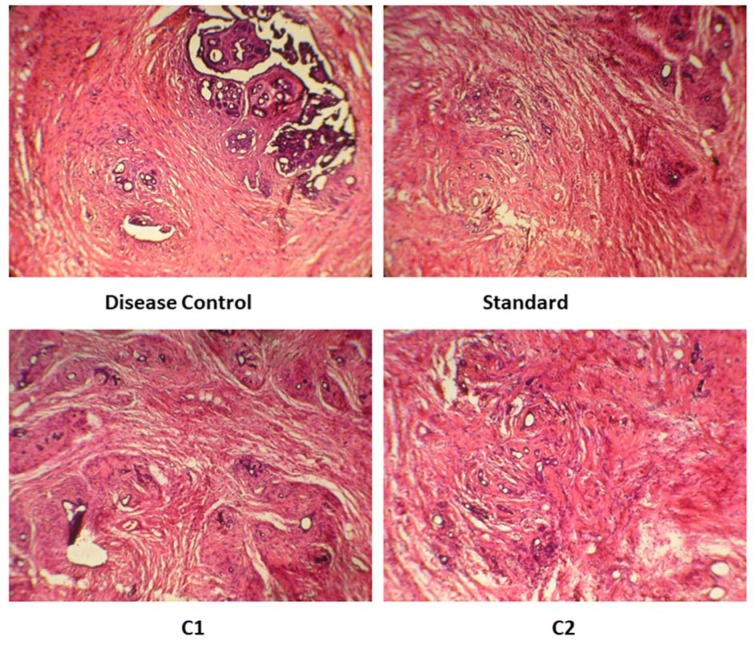
Histology of breast tumor

Histopathology of tumor in MNU control group exhibited a distortion in the normal architecture of rat mammary gland. Loss of tubuloalveolar pattern was observed along with the presence of large epithelial cells. In contrast, histopathology of the standard group displayed a restoration in the tubuloalveolar pattern. Treatment with C1 was not significant in comparison with the standard as observed by the distorted architecture of the epithelial cells. Moreover, C2 treatment was not able to restore the altered shape of the alveolar cells (Figure 8(Fig. 8)).

## Discussion

The present study was aimed to evaluate the anticancer potential of two 5-acetamido chalcones derivatives C1 and C2. They were tested for their cytotoxic potential by performing MTT assay (directly measures mitochondrial activity hence viability of cells) on MCF-7 and MDA-MB-231 with an incubation period of 48 h. C1 and C2 showed cytotoxicity with IC_50 _≤ 100 µM against both cancer cell lines. C2 was highly active whereas C1 was moderately active against MCF-7. Both the compounds were highly active in MDA-MB-231 cell line when compared to MCF-7 cells at micromole concentration. MCF-7 was selected for further study to correlate the mechanism of the synthesized compounds in MNU induced tumors, an ER+ve breast carcinoma model.

*In vitro* mechanistic study for the determination of apoptosis in MCF-7 cells was performed by dual staining (AO/EB) to find out the induction of nucleomorphological changes by the synthesized compounds. Tumor-selective cell death is the goal of cancer therapeutics and apoptosis as a mechanism of cell death offers therapeutic advantages (Sellers and Fisher, 1999[20]). Nucleomorphological changes significantly (p<0.05) increased in all treatment groups as compared to normal control. In MCF-7 cells treated with C1 and C2, a change in cellular morphology, including chromatin condensation and nuclear fragmentation which is a characteristic feature of apoptotic cell death was observed suggesting the apoptosis-inducing potential of the compounds. 

Metastatic spread of cancer cells from the primary site to distant organs is a major concern in cancer therapeutics (Khan and Mukhtar, 2010[13]). Scratch wound assay was performed to determine the anti-migratory potential of the test compounds. A significant reduction in the migration of MCF-7 cells was observed in C1 and C2 groups after 24 h and 48 h of treatment as compared to control. Our observation suggests the anti-migratory potential of the test compounds which is a property of anti-cancer therapies. 

MNU (N-methyl-N-nitrosourea) induced breast carcinoma is an *in vivo* model which mimics ER+ve breast cancer (McCormick et al., 1981[17]). In the present study, we evaluated the synthesized compounds C1 and C2 against MNU induced breast cancer. A quantitative method of estimating tumor progression in breast cancer model involves the determination of tumor volume (Faustino-Rocha et al., 2013[7]). Change in tumor volume was monitored weekly using a digital Vernier caliper. We observed a decrease in tumor volume in the treatment group compared to MNU control suggesting their ability to halt tumor progression. Organ indices are markers of toxicity (Vaijayanthimala et al., 2012[22]), which were calculated for liver, kidney, heart, and spleen. In all treatment groups, there was no significant difference in liver, spleen, heart and kidney index suggesting the safety of the compounds towards the major organs. Hematological estimations were performed to evaluate the effect of C1 & C2 on blood parameters. No significant changes were seen in the treatment groups as compared to MNU control group on the blood parameters. 

Liver function test (AST/ALT) and kidney function test (Creatinine) were also performed to check the toxicity of selected chalcones on organ functions. As per the results, doxorubicin and C1 treatment showed a significant decrease in AST level compared to MNU control group which is not considered as toxic sign clinically. No other changes were observed in evaluated parameters. Thus it can be concluded that C1 and C2 did not have any toxic effect on liver and kidney function. 

In pathological conditions like cancer, an increase in endogenous antioxidant level can prevent the damage associated with free radical generation (Pham-Huy et al. 2008[18]). Effect of C1 & C2 on endogenous antioxidants were evaluated in tumor and liver homogenate. In tumor samples, significant (p<0.05) increase was observed in SOD levels for all treatment groups, an increase in GSH level only in C2 treatment group while an increase in MDA level in Doxorubicin and C1 treatment groups. Thus overall effect showed C2 possessed better antioxidant potential compared to other treatment groups suggesting its potential in altering the endogenous antioxidant levels. Furthermore, we also observed an increase in the endogenous antioxidant levels in liver homogenate, where SOD levels were increased in doxorubicin treatment group, increase in GSH with C2 treatment, and increase in MDA level by doxorubicin, whereas a decrease in the level of MDA in the C2 treatment group was observed suggesting a protective effect against lipid peroxidation. 

Moreover, an induction of apoptosis is evident with the formation of a small fragment of DNA which can be observed as a ladder by gel electrophoresis. In tumor homogenate, we observed the formation of DNA ladder by this technique suggesting the induction of apoptosis by C1 and C2 treatment.

## Conclusion

In the present study, two 5' acetamido chalcone derivatives were synthesized and evaluated for their anticancer potential in breast cancer. Based on *in vitro* studies such as cytotoxicity study, anti-oxidant study and *in vivo* studies in MNU model such as evaluation of tumor volume, tumor weight and endogenous anti-oxidant we can conclude that C1 and C2 possess anti-cancer potential with anti-oxidant activity.

## Figures and Tables

**Table 1:**
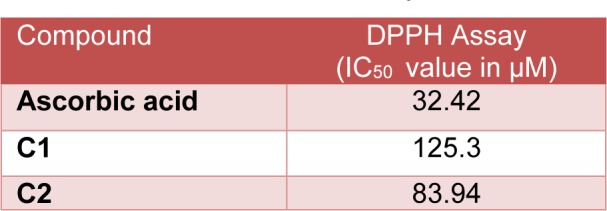
*In vitro* antioxidant assay

**Table 2:**
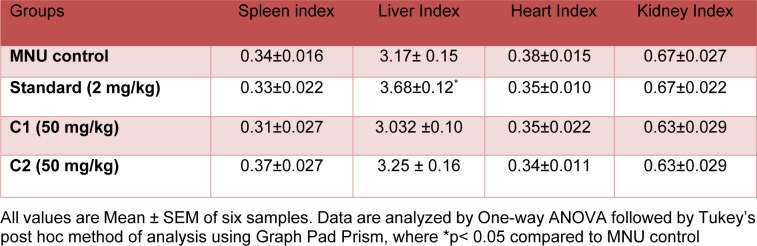
Effect of C1 & C2 on organ index

**Table 3:**
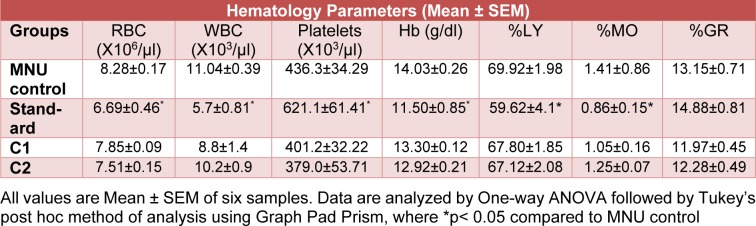
Effect of treatment C1 and C2 on the blood cell count in MNU induced breast cancer model

**Table 4:**
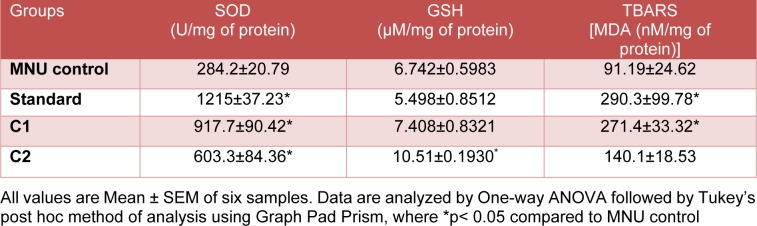
Tumor endogenous antioxidant status

**Table 5:**
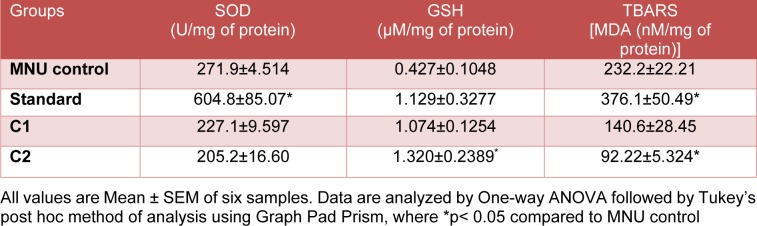
Liver endogenous antioxidant status
